# Tracking hematopoietic stem cells and their progeny using whole-genome sequencing

**DOI:** 10.1016/j.exphem.2020.01.004

**Published:** 2020-03

**Authors:** Henry Lee-Six, David G. Kent

**Affiliations:** a Wellcome Trust Sanger Institute, Wellcome Genome Campus, Hinxton, United Kingdom; b York Biomedical Research Institute, Department of Biology, University of York, York, United Kingdom; c Wellcome MRC Cambridge Stem Cell Institute, University of Cambridge, Hills Road, Cambridge, United Kingdom; d Department of Haematology, University of Cambridge, Cambridge, United Kingdom

## Abstract

Despite decades of progress in our understanding of hematopoiesis through the study of animal models and transplantation in humans, investigating physiological human hematopoiesis directly has remained challenging. Questions on the clonal structure of the human hematopoietic stem cell (HSC) pool, such as “how many HSCs are there?” and “do all HSC clones actively produce all blood cell types in equal proportions?” remain open. These questions have inherent value for understanding normal human physiology, but also directly inform our comprehension of the process by which the system is subverted to drive diseases of the blood, in particular blood cancers and bone marrow failure syndromes. The critical link between normal and abnormal hematopoiesis is perhaps best illustrated by the recent discovery of clonal hematopoiesis in healthy people with no abnormal blood parameters. In such individuals, large clones derived from single cells are present and are dominant relative to their normal counterparts, but their presence does not necessitate abnormal blood cell production. Intriguingly, however, these individuals are also at a significantly greater risk of developing leukemias and of cardiovascular events, underscoring the importance of understanding how blood stem cell clones compete against each other.

## Highlights

• Using acquired somatic mutations as a genetic barcode allows reconstruction of a stem cell “family tree” of relatedness.
• Understanding HSC clonal dynamics is central to understanding how clones emerge to drive leukemias and other diseases.
• Many HSC clones, most with multilineage output, make blood.
• HSCs acquire mutations linearly over life, but more slowly than other tissues.
• There is now a vast potential for understanding the relatedness and origin of various cell types across multiple adult tissues.

Despite decades of progress in our understanding of hematopoiesis through the study of animal models and transplantation in humans, investigating physiological human hematopoiesis directly has remained challenging. Questions on the clonal structure of the human hematopoietic stem cell (HSC) pool, such as “how many HSCs are there?” and “do all HSC clones actively produce all blood cell types in equal proportions?” remain open. These questions have inherent value for understanding normal human physiology, but also directly inform our comprehension of the process by which the system is subverted to drive diseases of the blood, in particular blood cancers and bone marrow failure syndromes. The critical link between normal and abnormal hematopoiesis is perhaps best illustrated by the recent discovery of clonal hematopoiesis in healthy people with no abnormal blood parameters [1, 2, 3]. In such individuals, large clones derived from single cells are present and are dominant relative to their normal counterparts, but their presence does not necessitate abnormal blood cell production. Intriguingly, however, these individuals are also at a significantly greater risk of developing leukemias and of cardiovascular events [4,5], underscoring the importance of understanding how blood stem cell clones compete against each other.

**Figure 1:**
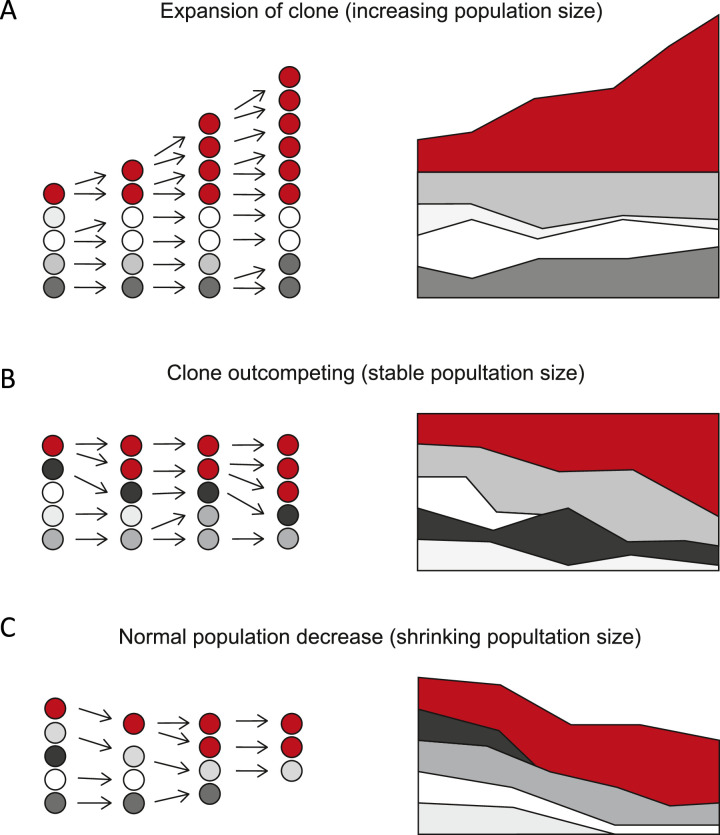
Numerous possible routes to clonal hematopoiesis. **(A)** Model A depicts what would happen if the clone in question has expanded in size in an independent fashion (i.e., increased self-renewal) without affecting or being affected by the normal stem cell pool, thus increasing the total number of stem cells. **(B)** Model B depicts what would happen if the clone actively competes with the normal stem cell pool to take up a larger share of the total stem cell number without actually changing that number. **(C)** Model C depicts what would happen if the stem cell pool is decreasing in size, but the clone in question displays greater resilience and represents a greater proportion of the total stem cell pool not because it has expanded greatly, but rather because the other stem cells have been depleted more (i.e., the denominator shrinks). In all cases, a static measurement of the clone would show an ∼60%–70% contribution of the clone but the cellular mechanism would be very different.

To determine what is abnormal, we first need to understand the range of clone size distributions that should be considered “normal.” Does clonal hematopoiesis simply represent the detectable tail of a distribution of clone sizes across the whole population (in the same way that patients with high blood pressure are the tip of a normal distribution of blood pressure), or does clonal hematopoiesis represent a qualitatively distinct state? The presence of somatic mutations in genes that are also found in myeloid malignancies (e.g., *TET2, DNMT3A*) suggests the latter, but a number of possible scenarios could explain their existence (Figure 1).

As clonal hematopoiesis is covered in other articles in this collection [2,3,6], we focus this review on recent studies that have used whole-genome sequencing to track clonal dynamics in unperturbed human hematopoiesis and touch upon future applications of the approach in studying how aberrations in clonal dynamics are evidenced in disease.

## Methods to track HSCs

Studying the behavior of stem cell clones requires a way of tracking them independently of one another. A considerable number of approaches in animal models or in humans undergoing HSC transplantation have been devised, dating back to the use of chromosomal [7] and enzymatic [8] markers to affirm clonal origin [9]. Using irradiation to induce traceable clonal marks, Becker et al. [10], Siminovitch et al. [11], and Wu et al. [12] serially transplanted bone marrow cells from mice and were able to demonstrate formally that the same clonal unit could generate cells of both the myeloid and lymphoid lineages. The whole-genome sequencing-based approaches discussed later in this review, which use somatic mutations to track cells, hark back to these original experiments using chromosomal markers.

A more experimentally tractable method of following multiple clones was developed in the 1980s in which cells labeled with retroviral barcodes or by unique viral integration sites were transplanted into irradiated recipients [13,14]. Barcodes could be found in all lineages, after transplantation into a second mouse, they could be found again, indicating that the transplanted cells were primitive and capable of creating daughter stem cells [15]. In these transplantation experiments, only a handful of stem and progenitor cells were found to be responsible for the majority of the blood produced in the salvaged animal [16,17], and these efforts culminated with the remarkable result that transplantation of even a single mouse [18] or human [19] HSC *could* be sufficient to reconstitute long-term multilineage hematopoiesis in mice. That a small number of HSCs *can* reconstitute long-term multilineage hematopoiesis in the transplantation setting, however, does not mean that such a small number of HSCs are actually driving the entirety of hematopoiesis in a physiological setting. Moreover, when large numbers of marked hematopoietic stem and progenitor cells are transplanted into animals, blood production remains polyclonal over long periods, with hundreds to thousands of unique markers detected years after the transplant 20, 21, 22.

Tracking cells via transplantation is associated with the disadvantage that the stress associated with the conditions of transplantation may affect the way that the transplanted cells behave. Certain cells that behave physiologically as HSCs may not do so in transplantation, and vice versa, or the dynamics of the system may be perturbed. Indeed, experiments in mice comparing in vivo labeling with transplantation suggest that only a fraction of the cells that would have behaved as HSCs would have engrafted successfully in vivo [23].

Recent work exploiting advances in inducible genetic labeling in mice to mark HSCs in vivo have begun to address these issues [24]. One of these approaches used an inducible sleeping beauty transposon system to label cells uniquely at a specific timepoint [25,26]. This technique marks stem and progenitor cells in vivo, allowing some of the first high-resolution insights into unperturbed hematopoiesis. Months after labeling, when the shortest-lived progenitors will have exhausted, transposon tags were detected in peripheral blood fractions. Tags were frequently shared across different differentiated blood cell types, indicating that—in unperturbed mice—most of the marked cells had multilineage outputs [26]. Longitudinal follow-up revealed that tags were rarely shared across different timepoints. One explanation for this is that different stem cell clones take turns producing blood one after another (i.e., clonal succession) [27]. A potentially more plausible explanation is that there are so many HSC clones that the limited sampling of peripheral blood would be unlikely to find the same tag across multiple timepoints, even if the tag were always present in blood cells across all timepoints. A second sophisticated method of marking cells uniquely in vivo used a polylox recombination system to generate random combinations of molecular cassettes [28]. The combinatorial diversity is such that the same sequence of cassettes is highly unlikely to occur in different cell lineages, thereby enabling the labeling of embryonic cells when HSCs first emerge. After birth, many barcodes were detected, indicating that the adult HSC compartment is a mosaic of at least hundreds of embryonic clones, again mostly with multilineage output. As multiple adult HSC clones are nested within embryonic clones, the number of embryonic clones forms an extreme lower bound for the number of active HSCs. Inferences about HSC clonal structure can also be drawn from population-level analyses [23,29], which have also supported the notion of large numbers of HSCs contributing to hematopoiesis. After permanent induction of the expression of yellow fluorescent protein in 1% of immunophenotypic HSCs and all their descendants, limiting dilution analysis estimated that ∼30% of immunophenotypic HSCs contributed to hematopoiesis over a mouse's lifetime [23]. Perhaps one of the most important contributions of these studies was the evidence that bone marrow transplantation, the gold standard HSC functional assay, vastly underestimates the functional output and mature cell production of multipotent progenitor cells [23]. This does not mean that HSCs are not ultimately responsible for the maintenance of day-to-day hematopoiesis, but it does suggest that the gap between HSC and multilineage progenitor with respect to clone durability might not be as large as previously postulated. Overall, these studies of unperturbed hematopoiesis in animal models combined to suggest that daily hematopoiesis is sustained by large numbers of multipotent cells, but quantifying these numbers exactly remains challenging.

## Transitioning to human blood

Sixty-five million years of evolutionary divergence, coupled with the long life span and large size of humans, make it difficult to extrapolate from mouse studies to estimate human stem cell numbers and dynamics. Furthermore, mice are typically studied under pathogen-free conditions, the proportion of peripheral blood cells that are myeloid in humans is larger, and there are many known differences between HSCs from the two species, including immunophenotypic definition, cytokine requirements, and differences in the bone marrow niche 30, 31, 32, 33.

Studying human hematopoietic stem cell function and clonal dynamics, however, comes with its own significant experimental challenges. Historic work has focused on xenotransplantation of tagged human cells into animals. These studies face all the caveats of autologous and allogeneic transplantation, with the added complication of differences between the human niche and that of the recipient immunocompromised animal. Limiting dilution transplantation experiments support the idea that thousands of bone marrow cells have the potential to act as stem cells 34, 35, 36. Estimates of HSC number from these approaches were divergent, which may be explained by differences in how significantly immunocompromised the animals were, the transplantation regimen, and the amount of ex vivo manipulation of stem cells [37].

Notwithstanding the technical and ethical challenges, progress has been made in directly assessing human HSCs off the back of gene therapy trials by using the unique genetic insertion site of the therapeutic vector as a trackable clonal marker. These trials have provided the first opportunity to study directly the transplantation and relative competitive ability of HSCs in people. Despite the fact that analysis of viral insertion sites is only semiquantitative, counting the number of unique insertion sites detected years after transplantation has provided a lower bound on the number of active stem cells in the transplantation setting. Long-term multilineage hematopoiesis has been demonstrated in these patients [38,39], with at least hundreds [40] to thousands [39] of stem/progenitor cells contributing over 2 to 4 years of follow-up.

As in the mouse, though, transplantation represents a nonphysiological setting where HSCs demonstrate their potential rather than their behavior in a normal unperturbed setting. Methods for studying native human hematopoiesis have been based on the detection of markers that vary naturally between different somatic human cells. In women heterozygous for an X-linked marker gene, detection of the proportion of cells that have inactivated either X chromosome provides some insight into population dynamics. In the absence of significant selection and/or genetic drift, half of the cells should express each X chromosome. Small imbalances, or “XCI skewing,” that emerge with neutral genetic drift or with selection allowed the first inferences based on binomial statistics, which estimated a minimum of 400 active HSCs [41]. Later work in elderly patients revealed an increase in XCI skewing with age, and this was used to infer the rate of clonal drift during aging [32].

Taken together, these studies across humans reinforce the work from animal studies that a large number of multilineage clones drive blood production at steady state, with the most physiological models providing lower bound estimates of stem cell numbers. Notwithstanding these significant advances, little is known about unperturbed human hematopoiesis, largely because of the absence of techniques to track large numbers of clones individually.

## Using spontaneous somatic mutations as natural barcodes

Somatic mutations occur in normal human cells over the course of life, are inherited stably by their descendants, and can be reliably detected by DNA sequencing; the vast majority have no phenotypic effect. They can therefore serve as excellent clonal markers. Early evidence from exome sequencing of blood colonies indicated that mutations accumulate approximately linearly over time, and at a sufficiently high rate that whole-genome sequencing of a blood cell should reveal multiple potential clonal markers [42]. Uncovering the mutations present in individual blood cells for use as clonal markers has become feasible in recent years because of the development of reliable methods for culturing single hematopoietic stem and progenitor cells (HSPCs) into large colonies that produce enough DNA, as well as the significant reduction in costs of sequencing a whole genome.

## Whole-genome sequencing to track somatic mutations

Two studies have so far exploited whole-genome sequencing to track hematopoiesis in unperturbed healthy humans [43,44]. Both followed broadly the same experimental workflow of isolating single stem and progenitor cells by fluorescence-activated cell sorting (FACS) and expanding them in vitro into sufficiently large clonal populations to permit whole-genome sequencing with good coverage. They then assayed different peripheral blood cell types for the mutations that had been discovered by whole-genome sequencing. In effect, this is similar to the inducible cell-tagging systems discussed above, but with somatic mutations as unique clonal tags. An important conceptual difference is that, unlike inducible cell-tagging systems, which induce tags at a given timepoint and then follow them prospectively over the course of the experiment, somatic mutation studies assess clonal dynamics retrospectively, with mutations that have accumulated over time being discovered and assessed from the end of the time course of a natural experiment. Furthermore, because the mutation “tags” are acquired continually over the course of life rather than at one timepoint, additional information is available from observing which tags are nested within other tags, which informs on the clonal structure of the population of cells. The cell types that are whole-genome sequenced are an important consideration (Box 1).

Box 1 **Cell type matters, but not as you might expect**

*Sequencing any blood cell reveals mutations that mark stem cell clones*

**Figure 2:**
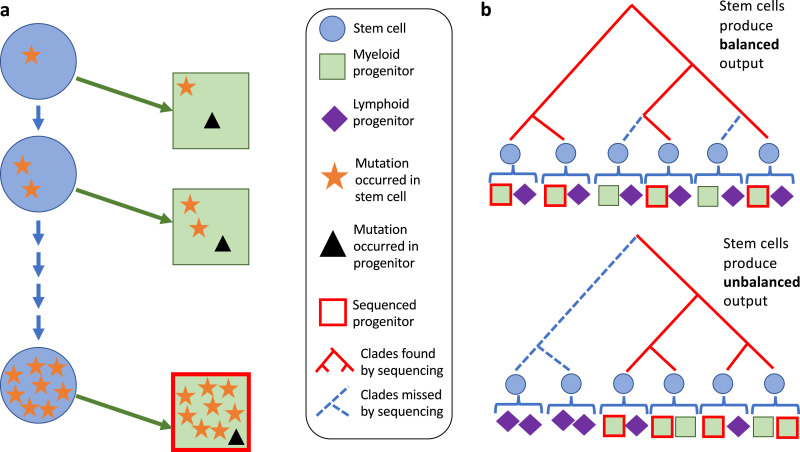
Effect of cell types that are sequenced on studies. **(A)** Sequencing progenitors reveals mutations that occurred in stem cells. Mutations that occurred in stem cells accumulate over time. When a progenitor from an adult is sequenced, most of the mutations discovered occurred in stem cells. **(B)** The cell types that are sequenced may affect the phylogeny that is reconstructed if stem cells do not produce balanced output. In the top phylogeny, all stem cells produce both myeloid and lymphoid cells. Sequencing only myeloid progenitors samples the phylogeny evenly, such that an accurate representation of the phylogeny is reconstructed. In the bottom phylogeny, some stem cells produce disproportionately more lymphoid than myeloid progenitors. Sequencing only myeloid progenitors undersamples parts of the phylogeny that are biased toward lymphoid cells.

Both studies whole-genome sequenced colonies grown from primitive multipotent stem and progenitor cells. Counterintuitively, sequencing a progenitor reveals mostly mutations that occurred in a stem cell. This is because progenitors only relatively recently stopped being stem cells. In other words, the vast majority of mutations that are discovered in a progenitor will have occurred in a cell that was behaving in vivo in a manner that fits the conceptual definition of a stem cell. To illustrate this concept, imagine whole-genome sequencing a progenitor from a 60-year-old man. Even if this progenitor is relatively long lived, it will retrace its origin to a stem cell that existed recently (e.g., 1 year ago). Assuming a constant mutation rate from ages 0 to 60, 98% (59/60) of mutations will have occurred in an ancestral stem cell. These mutations will therefore serve as clonal markers for the progeny of stem cells, whether they are actually functionally assayable stem cells or not (Figure 2A).

*The mutations detected may not be representative of the whole stem cell pool*

The cell types that are sequenced matter much more if stem cell clones produce biased output. This is because they affect the way in which the stem cell pool would then be sampled. If, say, only neutrophils were whole-genome sequenced, and some clonally related subset of the HSC pool were biased toward producing them, clonal markers of this subset of HSCs would be overrepresented in the experiment (Figure 2B). To limit this effect, both studies sequenced different myeloid progenitors (in addition to immunophenotypic HSCs). Despite the attempt to minimize any skewing in reporting the complete HSC population, it must be highlighted that the fractions isolated across both studies (HSCs, multipotent progenitors [MPPs], granulocyte–monocyte progenitors [GMPs], megakaryocyte–erythroid progenitors [MEPs], and common myeloid progenitors [CMPs]) contain more myeloid than lymphoid progenitors in the “non-HSC” portion of cells. This may therefore result in an underrepresentation of stem cell clones that contribute to lymphoid cell production. This was partially addressed in one study by surveying mature lymphoid cells longitudinally to demonstrate that many branches contributed to B lymphopoiesis. The data on T lymphocytes, however, (where many clones were underrepresented compared with granulocytes and B lymphocytes) suggests the possibility that the branching structure may not fairly represent the entirety of blood cell production.

Thus, the choice of cells to whole-genome sequence does not matter in terms of the level in the differentiation hierarchy at which their mutations occurred: sequencing any blood cell will reveal mutations that occurred in stem cells. Nonetheless, the choice of sequenced cells could affect our understanding of stem cell biases in producing certain cell types: If we do not sequence T lymphocytes we may miss stem cell clones that are biased toward producing them.

Alt-text: Unlabelled box

The detection of somatic mutations is subject to both false-positive and false-negative errors. The mutation detection methods used in both studies were previously published [45,46], and several experimental results support a high specificity of the mutation calling methods. First, the majority of mutations with a high allele fraction that were discovered by whole-genome sequencing were also detected in the recapture phase of the experiments. Second, the mutation burden and mutational signatures were consistent from cell to cell and between the two experiments, implying that neither approach dramatically skewed the type of mutation being called. Third, the mutation burden increased linearly with the age of subjects. The conservative mutation calling approaches in both studies may, however, have resulted in false-negative mutation calls. That said, as long as false-negative errors are evenly distributed (e.g., that mutations that occur early in life are equally likely to be missed as those occurring later), a low sensitivity should not skew analyses of clonal dynamics.

## Depth versus breadth: Complementary studies

Lee-Six et al. [43] took the approach of studying one individual in great depth, a healthy 60-year-old man, from whom 140 single immunophenotypic HSPCs were isolated by flow cytometry, grown in culture, and then whole-genome sequenced. Most of these HSPCs were derived from a bone marrow aspirate, but some were from peripheral blood, to detect and avoid bias that might result from spatial clustering of similar cells in the bone marrow. In contrast, Osorio et al. [44] opted for breadth over depth, whole-genome sequencing 22 HSPCs from seven healthy donors, ranging in age from 0 (umbilical cord blood) to 63 years.

## Detecting genomic mutations in other blood cells

The methods by which the set of somatic mutations (identified in the initial whole-genome sequencing) were detected in other blood cell fractions also differed. Lee-Six et al. [43] designed a bait set that included >7,000 mutations that had been discovered in the initial sample set and used it for ultradeep-targeted sequencing (range: 268 × to 4669 ×) of bulk peripheral blood cell fractions across different timepoints. Osorio et al. [44] genotyped 125 further colonies and analyzed the presence or absence of 13 mutations in different blood cell fractions from one patient. Despite these differences in design, the findings of the two studies (Table 1) are similar. Below we discuss their implications.

**Table 1:** Common findings from the two studies

	Lee-Six et al. [43]	Osorio et al. [44]
Clonal contributions to blood production	50,000–200,000 stem cells make granulocytes at any one time.	Each of 13 clonal markers were found in differentiated blood cells.
Lineage output of clones	The majority of HSC clones responsible for myeloid blood production also made B lymphocytes, but not T lymphocytes.	HSC clones were ancestral to all of granulocytes, erythroblasts, megakaryocytes, and B cells.
Embryonic specification of blood	The common ancestor of blood occurs before gastrulation. The daughters of this common ancestor contribute unequally to blood.	The daughters of the common ancestor of blood contribute unequally to blood.
Mutation rate and patterns of HSCs	Based on one patient, mutation rate is ∼17 mutations per year. Blood mutations occur in a characteristic trinucleotide context.	Based on patients of different ages, mutation rate is constant and occurs at 14.2 mutations per year. Mutational signature analysis reveals a signature of blood production with features consistent with the trinucleotide context of Lee-Six et al. Its transcriptional strand bias suggests that it is due to guanine adducts.

## Inferences about the development of blood

**Figure 3:**
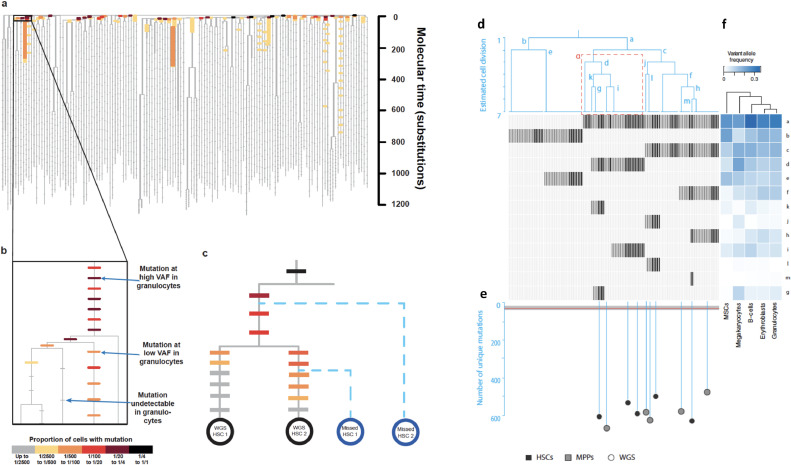
Phylogenies from (A–C) Lee-Six et al. [43] and (D–F) Osorio et al. [44]. **(A)** The phylogeny of cells is shown in gray, with branch lengths proportional to the numbers of somatic mutations (y-axis). Each tip of the phylogeny leads to a stem or progenitor cell that has been whole-genome sequenced. Information from targeted sequencing of peripheral blood granulocytes is overlain. This is shown more clearly in the inset **(B),** which zooms in on one portion of the tree. On top of the underlying structure of the phylogeny (gray) are placed *horizontal bars*. Each bar represents a mutation in the bait set for targeted sequencing. The bars are colored according to the proportion of cells in the sample that carry the mutation, indicated by the color scale. Undetectable mutations are colored gray and shown as smaller bars. Mutations are assigned to a branch based on the colonies in which they are present. **(C)** This schematic explains that the allele fractions of targeted mutations in peripheral blood decline down the branches because of undetected coalescences with stem cells that were not whole-genome sequenced, but are producing granulocytes. **(D)** The phylogeny of whole-genome sequenced clones. Branches are labeled a–m, each of which represents a mutation that defines the lineage. The presence (hematopoietic stem cells: *black*, multipotent progenitors: *dark gray*) or absence (pale gray) of each mutation in genotyped clones is shown in the panel below the phylogeny. **(E)** Continuation of the phylogeny for whole-genome sequenced clones, with branch lengths proportional to mutation load. **(F)** Allele fractions of mutations a–m in mature blood cell populations.

Both studies used the somatic mutations discovered in individual HSPCs to build a family tree of how the cells were related to one another. Figure 3 illustrates the structure of the phylogenetic trees from both studies. The smaller phylogeny of Osorio et al. [44] was constructed manually by inspecting the pattern of mutation sharing between cells. The phylogeny in Lee-Six et al. [43] was reconstructed computationally using methods that aim to identify the tree that is most likely to produce the observed set of combinations in which somatic mutations are shared between different individual cells, based on a model of how somatic mutations are acquired and detected. The phylogeny was then validated using orthogonal methods (the range of methods that can be used to construct phylogenies is discussed in Yang and Rannala [47]). In each case, the majority of the branchpoints in the phylogenies occur at the top of the tree. As you look up toward the root of the phylogenies, you are looking back in time, and the earliest branchpoints tell us about cell divisions that occurred in the embryo. That most branchpoints in both trees occur early is probably due to that fact that when a relatively small sample is drawn from a large population, cells are likely only to be distantly related and so will share only embryonic branchpoints. A rapid population expansion in the embryonic phase, or other population size changes over life, may also contribute to this effect, however. Both studies found the mutations on these earliest branches also to be present in cells from other germ layers (mesenchymal stem cells in Osorio et al. [44], buccal epithelium in Lee-Six et al. [43]), indicating that these mutations and branchpoints must occur early in embryogenesis. The daughters of the very first were found to contribute unevenly to blood, with one daughter ancestral to approximately two-thirds of the sampled cells and one ancestral to approximately one-third of the sampled cells. This supports similar findings in the mouse [47] and in bulk sequencing of human blood [48]. The Lee-Six et al. [43] study found that the mutation on one side of the first division that was present in two-thirds of blood cells was also present in two-thirds of cells from the buccal swab, and the mutation on the other side present in one-third of blood cells was present in one-third of buccal cells. This indicates that the common ancestor of blood was the same as the common ancestor of buccal cells, which derive from another germ layer, a finding that indicates that this common ancestor must have existed very early in development, perhaps as far back as the fertilized egg.

## Estimating HSC numbers

**Figure 4:**
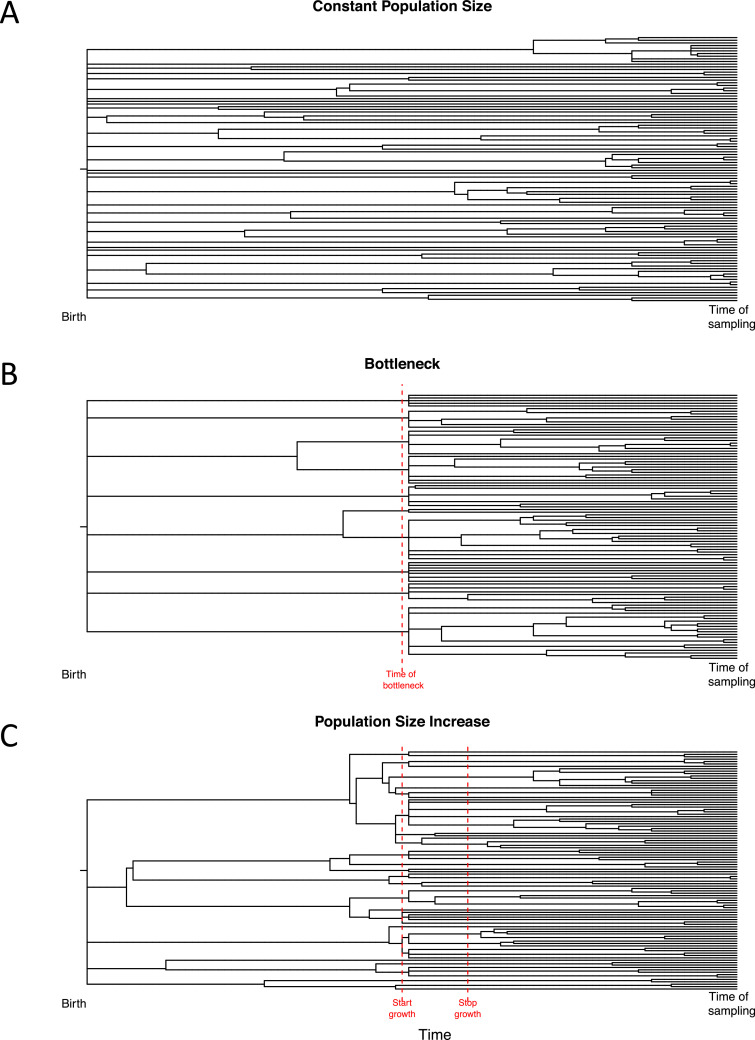
Simulations illustrate that phylogenies reflect the population size trajectory. All simulations were produced using a Fisher–Wright model of neutral drift. At the end of each simulation, 100 cells are sampled and their phylogeny reconstructed. **(A)** A constant population size. **(B)** A sudden strong bottleneck in population size at the point in time indicated by the *red dashed line*. **(C)** An increase in population size between the *red dashed lines*, which stabilizes thereafter.

The size of the family tree of cells in one study permitted further analysis of stem cell dynamics by borrowing methods from population genetics. In epidemiology, the pattern of branching in a phylogeny of influenza genomes can be used to reconstruct the population history of the virus, showing that the population size increases in winter and drops in summer [49,50]. A phylogeny of a constant population size exhibits an expected distribution of branchpoints. If the population size increases, branchpoints increase in density around the time of the increase, and if there is a bottleneck, branches are pruned from the tree, resulting in a coppiced appearance (Figure 4). One of the attractive features of data sets generated by whole-genome sequencing is that methods developed for the fields of epidemiology, ecology, and population genetics can each be utilized.

When a phylodynamic inference program is applied to the tree structure of cells, a logarithmic increase in population size is observed early in life, consistent with the rapid growth of the HSC pool needed during embryogenesis and early childhood, followed by deceleration in the rate of increase, reaching a plateau in adulthood. If the mutation rate per year in HSCs is higher during development, as it may conceivably be to expand the stem cell pool, then this plateau will be reached before adulthood. The immunophenotypic stem cell pool (Lin^−^CD34^+^CD38^−^CD90^+^CD45RA^−^) has been reported to increase with old age [51], but, as has been suggested in mice, the functional stem cell pool may not [52,53]. Phylodynamic methods should report on the effective population size, which relates to the stem cell pool that is able to self-renew. It remains to be seen whether phylodynamic methods support an increase in HSC population size over the age of 60. This could be tested by repeating the study in older individuals.

## Measuring persistent clonal contribution to mature cell populations

Detecting clonal markers in differentiated blood cell types reveals which stem cell clones have progeny at the time of sampling. If only a subset of clones were making granulocytes, only the markers of those clones would be detected in the deep sequencing data of granulocytes. However, both studies found that markers from multiple nonnested clones were present in differentiated blood cells from each given time point (Figure 3). In other words, the production of blood is highly polyclonal, deriving from a large number of HSCs. If clonal markers are arranged on the phylogenetic tree (Figure 3A–C) one can see that mutations higher up the tree (i.e., those that occurred earlier in time) are present in a greater proportion of HSCs. This makes sense, as mutations lower down the tree occurred in nested clones: they are necessarily present in fewer cells. Rapidly, as one progresses down the tree, the mutations are present in such a small proportion of cells that they are no longer detectable, even with deep sequencing. This means that the mutations in early clones that *are* detectable will be present in many descendants, and so blood must be produced by many more stem cells than the number of clones apparent in Figure 3.

With some assumptions and a relatively simple model of hematopoiesis (Box 2), one can use the targeted sequencing data to estimate the number of HSCs that sit at the bottom of the tree and are ancestral to the blood sample. This is explained more formally in Lee-Six et al. [43], but in brief, the estimate comes from comparing targeted sequencing counts of each mutation in different granulocyte samples taken at the same time from the same individual. The number of mutant reads in each of the granulocyte samples is a proxy for the number of cells in that granulocyte sample that carried the mutation. If one knows the number of granulocytes in each sample, and the number of them that carry the mutation, one can adapt the logic of capture–recapture methods to estimate the number of stem cells (similar to how ecologists estimate population size by tagging animals in the wild [capture] and then recapturing animals at a later point and asking what proportion of them are tagged). Imagine that only 10 stem cells contributed to blood at any one time, and we have 100,000 granulocytes in each of two samples. We would expect all mutations from all 10 stem cells to be found in both granulocyte samples. Now consider that one million stem cells make blood at any one time, and we still have 100,000 granulocytes in each sample. It is not possible for mutations that are private to each of the one million stem cells to be found in any given granulocyte sample, as the number of granulocytes is smaller than the number of stem cells. Under the framework of an approximate Bayesian computation in an attempt to recapitulate the complexity of human hematopoiesis and the experimental setup, it was possible to estimate that 50,000–200,000 HSCs make granulocytes at any one time.

Box 2 **Simulated model of hematopoiesis**

Lee-Six et al. [43] chose an approximate Bayesian computation approach to estimate the number of stem cells [71]. This involves simulating a model of hematopoiesis thousands of times, while changing the number of stem cells in each simulation and recapitulating the whole experiment *in silico* for each simulation. The number of stem cells for the simulations that produce results most similar to the observed data are considered to be most probable. The model was chosen to be as simple as possible:

1. The size of the stem cell pool was constant over adult life.
2. Individual stem cells within the population of total stem cells replicated stochastically over life, and their clonal dynamics approximated neutral drift. There was no selection in the model, and the pool was considered to be homogeneous (i.e., there were not more or less quiescent compartments).
3. In each cell division, each stem cell acquired a number of mutations drawn from the Poisson distribution. Mutation rate was constant over life.
4. All of the stem cells with which the simulation was concerned were ancestral to a similar number of granulocytes.
  A variable mutation rate per year during development could have affected estimates of stem cell number. To avoid this, only mutations that had occurred after 100 mutations of molecular time, and so after the phase of population expansion, were used in this analysis.

Alt-text: Unlabelled box

Considering the data from both articles, it therefore seems highly likely that hematopoiesis in humans is hugely polyclonal, as previously suggested by animal and transplantation studies, but perhaps to an even greater degree than had been anticipated.

## Multilineage hematopoiesis: Multipotent clones are dominant in humans in vivo

Detecting a mutational marker in multiple differentiated blood cell types means that the cell that acquired that mutation had descendants that were capable of producing all of these cell types. Both studies found mutations that were shared in multiple blood cell types. Osorio et al. [44] found four mutations that were not shared with mesenchymal stem cells (and so are likely to have occurred after gastrulation) but were each detected in multiple differentiated blood cell types, including combinations of granulocytes, megakaryocytes, erythroblasts, and B lymphocytes (T lymphocytes were not assayed) (mutations j, l, m, and g in Figure 3F). Thus, all hematopoiesis-specific clones that they could detect were multipotent.

Lee-Six et al. [43] assayed granulocytes and B and T lymphocytes for mutations found by whole-genome sequencing. Mutations that occurred very high up the phylogeny, and so occurred in the embryo or early life, were shared by all three cell types. Later mutations, including ones occurring sufficiently far down the phylogeny that they are likely to have occurred in early adulthood, were shared by both granulocytes and B lymphocytes, indicating an ongoing contribution of multipotent adult HSCs to B lymphopoiesis throughout life. These mutations were, however, commonly absent from T lymphocytes. Possible explanations for this absence include the following:

1. There may be clones that skewed toward the production of granulocytes and B cells but not T cells. This may be because the colonization of the thymus is a process driven by a relatively small proportion of cells.
2. It may reflect the longevity of the T-cell pool. If most T cells matured in the thymus earlier in life, the clonal markers found in the T-cell pool would represent the stem cells that existed many decades ago rather than those contributing today.

Whatever the explanation, the limited sampling of the HSC pool means that the presence of clones that produce all three cell types cannot be excluded. Furthermore, the studies’ sampling strategies mean that clones that produce only lymphoid cells are likely to be missed (Box 1). More extensive sampling—at the whole-genome sequencing stage—of HSCs and of different peripheral blood cell fractions, including T lymphocytes and their precursors, as well as repetition of the experiment in patients of different ages, may help to clarify the cause of the divergence in ancestry of B and T lymphocytes. Similarly of interest, Osorio et al. [44] found mutations that were at higher allele fractions in each differentiated blood cell type than in megakaryocytes and, conversely, mutations that were at higher allele fractions in megakaryocytes than in other differentiated blood cell types, suggesting that megakaryocytes might diverge early and be sustained by a specific set of HSC clones. Ultimately, all blood cell types may be analyzed in this way, and their comparison may help to elucidate clonal hierarchies of lineage restriction, by analogy to experiments in mice..

## Further genomic insights

As a useful by-product to clonal tracking, whole-genome sequencing of blood stem and progenitors reveals a wealth of information about their genomes, with implications for our understanding of normal blood cells and leukemias. For example, the analysis of individuals of different ages by Osorio et al. [44] revealed a linear mutation accumulation with age, at the rate of ∼14 base substitutions per year, consistent with the ∼1,000 mutations seen in a 60-year-old in Lee-Six et al.’s study and previous studies of normality and malignancy [42,54].

Interestingly, the mutation burden found from bulk sequencing myeloid leukemias is not elevated relative to normal cells. Indeed, the mean number of mutations per AML genome in a cohort with a mean age of 55 was just over 400 [55]. Some of the difference between normal and cancer may be due to different sequencing platforms, mutation calling algorithms, or contamination of the matched normal tissue with leukemic blasts (such that somatic mutations appear to be germline). Another, potentially more biologically interesting, explanation is that the most recent common ancestor of the leukemia may have existed a long time ago: if a single leukemic cell were sequenced, it might have the same mutation burden as a single normal cell, but bulk sequencing misses a large proportion of the subclonal mutations in the tumor [56]. Depending on the strength of the effect of the first three factors, comparison with normal data could indicate that the most recent common ancestor of many AMLs occurred decades before diagnosis.

Additional genomic insights come from analysis of the pattern of mutations across the genome, where so-called “mutational signatures” can provide clues to the etiology of the processes causing them [57,58]. Single-base substitutions can be divided according to the identity of the original and resultant base into six types: C>A, C>G, C>T, T>A, T>C, T>G. Certain processes are associated with an excess of one type over the other. For example, smoking is associated with C>A changes. Examining one base upstream and one base downstream of the mutated base (the trinucleotide context) provides further resolution. The substitutions found in normal blood [43,44], clonal hematopoiesis [59], and certain leukemia genomes [60] have a distinctive pattern of trinucleotide substitutions. Osorio et al. [44] deconvoluted these mathematically into three distinct processes: signatures 1 and 5, ubiquitous signatures that accumulate in a clocklike manner over life in most tissues [54], and a signature that had a transcriptional strand bias with more C>T mutations on the transcribed strand. If, as is usually thought to be the case, this is a result of transcription-coupled nucleotide excision repair, this signature would be consistent with the repair of damage caused by a guanine adduct. With further characterization and additional samples, mutational signatures will be of significance to our understanding of hematopoiesis because they may in some cases report time spent in specific microenvironments. For example, one could imagine that particular features of the bone marrow microenvironment might protect cells from certain mutational process or caused others and that time spent in the bone marrow (or away from it) would then be revealed by analyzing mutational signatures.

## Comparison with other tissues

In recent years, many nonhematological normal human tissues have been investigated by sequencing. In most cases, the principal insights have been to catalogue the burden and range of somatic mutational processes and to describe the presence and effect of different driver mutations that characterize different histologically normal human tissues 61, 62, 63, 64, 65, 66, 67, 68. In comparison to the other tissues studied so far, blood has a relatively low mutation rate, and the set of mutational processes acting in HSCs seems to be more restricted than in other tissues studied to date.. For example, the average mutation rate per year in colonic stem cells is at least twice that of HSCs, and the diversity of mutational processes is greater [46,65]. This may be related to stem cell division rates, cell-specific DNA repair mechanisms, and differences in mutational exposures between different stem cell environments.

Inferences about the stem cell dynamics of normal tissues may also be drawn from experiments on sequencing of solid tissues. Unlike in the blood, which presents the unique opportunity of relatively random sampling of cells because of its liquid nature, studying the clonal dynamics of solid tissues requires assumptions about how clones grow and shrink in space. Nonetheless, important insights may be gained with simple models of development and stem cell dynamics. Patterns of embryonic development may be mapped out through careful reconstruction of phylogenies and comparison with anatomical location of the sequenced cells [48,63,64]. The growth of clones may be detected by sequencing microbiopsies of tissues and estimating clone size from allele fractions [61,62]. In the colon, the crypt fission rate and the time to monoclonal conversion within a crypt may be estimated [65,69]. A better understanding of how cells distribute themselves within a tissue may be necessary, however, before solid tissues can be studied to the same resolution as the blood. In contrast, solid tissues have led the way in demonstrating that sequencing can reveal the effect of various stressors on clonal dynamics. Whole-genome sequencing of human liver [66] and lung [70] reveals the changes in stem cell architecture associated with alcohol and obesity for the liver and smoking for the lung, helping to elucidate how stem cells behave under different pressures. Similar studies will be possible in the blood and should be undertaken within the coming years to assess the resiliency of the system to various mutational processes.

## Concluding remarks

Somatic mutations provide a flexible and powerful way to investigate unperturbed hematopoiesis. The insights gained range from clonal structure and lineage outputs to insights into the embryonic development and mutational landscapes of the blood. Whole-genome sequencing as a method to track unperturbed human hematopoiesis is a natural extension of decades of work in animal models using different markers to follow cells and opens opportunities for having a more direct assessment of what HSCs actually do in vivo throughout aging. It has been made possible by technological advances, and will become increasingly tractable as the cost of sequencing declines further and the amounts of input DNA required fall, such that shorter in vitro amplification phases, and eventually no in vitro amplification phases, are necessary to detect mutations reliably. This will allow whole-genome sequencing of all blood cell types. A wide array of future experiments suggest themselves to exploit this technology. Small numbers of individuals have so far been studied, and the person-to-person variability in all of the described parameters remains to be explored before general conclusions can be made. As many hematopoiesis-related disorders present in old age, following patients longitudinally for decades will reveal all stages of the changes in clonal structure associated with clonal hematopoiesis and malignancies, including the discovery of multiple genetic ways to arrive at the same physiological or pathophysiological state. One could also imagine that defining the clonal landscapes of a background population and comparing it with those in the early stages of malignancy may allow the early identification of risk of malignancy in a more robust manner than age-related clonal hematopoiesis. Eventually it may even become possible to intervene to avert clonal outgrowths by supporting clones with a lower propensity to drive leukemia or targeting those that associate with malignancy. In all cases, more extensive cell sampling, sequencing different cell populations (e.g., mature lymphoid cells), and combining sequencing of genomes with that of transcriptomes could provide further information on exactly which clones produce which cells and how they are related to one another.
